# Action Research on Group Consulting of Family Legal Education for Adolescent Parents in China

**DOI:** 10.5539/gjhs.v4n5p125

**Published:** 2012-08-09

**Authors:** Ying Ge, Wei Feng

**Affiliations:** 1 School of Education of Chongqing University of Arts and Sciences, Chongqing, China; 2 School of Education of Southwest University, Beibei District, Chongqing, China

**Keywords:** adolescent parents, family legal education, group consulting, action research

## Abstract

**Aim:**

In this experimental study, we made an attempt to explore the approach and method to improve the legal cognition and family legal education level for adolescent parents.

**Methods:**

10 parents of students of grade two in a middle school of Chongqing in China were provided with group consulting and training. We adopted action research method to make overall assessment on the needs, execution and results of group consulting and training activity about family legal education for adolescent parents.

**Results:**

After educating intervention group training of action research, the legal cognition level and the mastery and utilization of family legal educational method of adolescent parents get rising.

**Conclusion:**

Through the assessment of action research, the group training manner is a useful group consulting manner to make family legal education for adolescent parents. The program was feasible; the method was effective; the intervention effect was obvious.

## 1. Introduction

In recent years, the juvenile crime rate keeps up; the crime types, means and consequences tend to be complicated and serious. One important reason is the failure of family education. Therefore, attachment of importance to family legal education will be a powerful line of defense against juvenile crimes.

Family legal education means the process of education given under family environment to minor children by their parents, through increasing legal knowledge, improving legal awareness and mastering legal education methods, to cultivate minor children’s legal awareness and law-abiding habits. For a long time, the research on the family legal education in China has been weak, lack of systematic empirical studies and with single research perspective; functions of family education have been disordered, and parents have been lack of legal knowledge and ability for legal education; and there have been no studies on education for parents on relevant juvenile laws (Ge & Feng, 2005). This research, therefore, in combination with the characteristics of physical and mental development of juveniles and the era characteristics of family development, studies the “parent counseling” in family legal education based on the law of legal norm learning, the group counseling and mental training. Action research is used to know about the needs of adolescent parents for improvement in legal cognition and methods of family legal education; the counseling contents and ways are discussed to formulate counseling program on parents’ legal knowledge and legal education method, and effectiveness of such program is assessed; the counseling is in group counseling manner, and the process and effectiveness of counseling are assessed, which provides a scientific basis and feasible measures for family legal education of a new period. In this way, the following hypotheses were tested: (1) the program of the group consulting will be feasible. (2) the method of the group consulting will effective. (3) the intervention effect of the group consulting will obvious.

## 2. Method

This research adopts action research method. The term of Action Research Method is coined by social psychologist Lewin in 1940s (Babbie, 2010). “Action research is a form of self-reflective enquiry undertaken by participants in social situations (educational situations) in order to improve the rationality and justice of their own social or educational practices, their understanding of these practices and the situations in which the practices are carried out.” (Husen, 1985). Based on the record of observation and action, it is carried out in a form of group counseling with mutual cooperation between group members in dynamic environment to change the actions which need to be changed through planned intervention and take recorded observation information as driving force of research development, which is more conducive to result inspection than pure theoretical research and is an important feature of action research method differentiating from pure theoretical research, belonging to qualitative research method (Druckman & Daniel, 2009). Action research is not a linear research process but a spiral periodic process (Hu & Chang, 1988). It is to practically solve problems by applying theories. During research, the planning, action and finding of action result are regarded as a circulatory process and through this dynamic circulatory process, the researcher and the participants communicate and share their experience. The reflection assessment of each stage is amended into the execution of next stage to continually perfect the program (Silverman, 2009). Hermeneutics is adopted for data analysis. Importance is attached to the participants on their explanation for these events and behaviors to form “understandings or views of participants” (Maxwell, 1996). The researcher fully communicate with the parents participated to obtain consensus on data interpretation in the interaction of parents, children and researcher.

In this research, therefore, importance is attached to the cooperation and participation of parents. The researcher acts as a planner, director and inducer to help parents to learn and think in a better way and encourage parents to participate into this research positively and actively to find and solve problems in actions and reflect and improve the content and execution method of educational intervention program with the researcher so as to improve the legal cognition level and application level of legal education methods of themselves.

**Figure 1:**
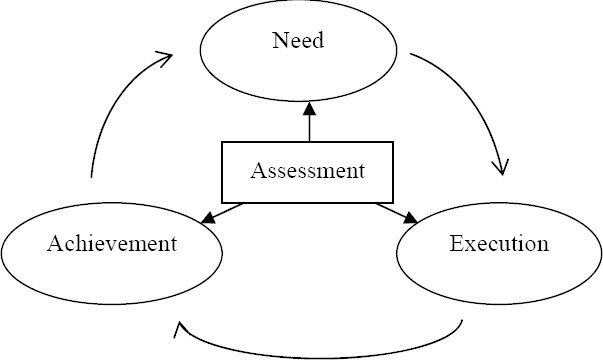
Assessment-centered Research Model Participants for this research are 10 parents of students of grade two in a middle school of Chongqing in China.

In the action research, “assessment” plays an important role (Lai & Kuo, 2008), and the assessment contents cover need, execution and achievement (as shown in Figure 1). “Need” refers to whether the parents desire and demand to participate in the education intervention for legal cognition and legal education methods; “execution” refers to the process of education intervention based on the program formulated by the researcher in accordance with parents’ requirements; and “achievement” is what parents gained upon the completion of education intervention. In the research, the researcher selected parents to cooperate with by putting forward questions; formulated and executed education intervention program on legal cognition and legal education methods for parents upon actual needs assessment; communicated with the parents and conducted assessment throughout the implementation of such program to establish partnership with each other; revised the collected feedback data on the program; and executed the program of the following unit in order to make the implementation of the program meet participants’ needs better. Upon the completion of all activities, assessment on effectiveness of such program was conducted, and interviews with relevant personnel, including the parents and their children, were conducted to support the assessment results. Data were collected by ways of record, note, questionnaire and interview (McNiff, Lomax, & Whitehead, 1996), and the interviews were fully recorded under the agreement of interviewees and then converted into words.

## 3. Procedures

### 3.1 Assessment on Needs for Education Intervention Program on Legal Cognition and Family Legal Education Methods for Adolescent Parents

Posavac & Carey (2006) think that it is of necessity to conduct needs assessment before the planning and design of a creative education activity program. Needs assessment generally aims at obtaining enough data and information to determine the detailed needs of counseling participants and whether the researcher is able to meet their needs on the one hand and to design appropriate activity program on the other hand (Liao, 1998).

#### 3.1.1 Expectations on Activities and Needs for Manners of Activities

In previous parents’ meeting, parents had expressed their expectations on expansion in legal knowledge, improvement in legal cognition level and capability to use various education methods through training.

Time and place for education intervention experiment. As parents have to work in the day, both parents and head teachers agreed that it was appropriate to conduct the intervention experiment at night instead of at weekends for the avoidance of affecting family life. Finally, the time determined for the intervention experiment was 18:30--20:30 on each Thursday for two months, and in total there were eight times. The place determined for the intervention experiment was the multi-media classroom where the students usually have classes. Such a place could give the parents sense of security as they were familiar with it.

#### 3.1.2 Name of Activities in Education Intervention Experiment

The original name of activities designed by the researcher was *Workshop for Growth of Adolescent Parents in Family Legal Education*. In the interview, parents expressed that in their eyes the word “growth” brought stress to them and they were afraid that others might see them in different lights. Therefore, the name of activities in education intervention in this research finally determined was *Workshop for Improvement of Adolescent Parents in Family Legal Education*.

#### 3.1.3 Manners of Activities in Education Intervention Experiment

The parents disliked formal teaching manner and being taught, instead, they liked group manner. If we adopt a relatively relaxed manner for education intervention, provide parents with chances to express their opinions and discuss together, and have uniform regulations for all members to abide by, parents’ rejection feelings can be significantly reduced.

Therefore, based on parents’ opinions, the researcher planned some flexible and diverse interaction activities in which discussion was emphasized through comprehensive application of case discussion, situation experience through role-play and sharing what have learned, and formulated a letter of acceptance for observation of group convention to increase confidentiality, safety and binding force. The above steps were taken to comprehensively plan the intervention program on family legal education for adolescent parents.

#### 3.1.4 Needs for Contents of Activity Program

With full consideration of all views, we found that parents’ needs for contents of education intervention program mainly include the knowledge concerning relevant laws and regulations on the protection of minors and the methods of legal education. Therefore, by reference to books and articles concerning legal education, family education, family psychology education counseling and program design for parent education (Cao, B-H Liu, & Y-G Liu, 2004; Yao & Feng, 2004; Fan & He, 2010; Miao, 2001; Liao, 2000; Liang & Tang, 2003), we have written the *Education Intervention Program on Workshop for Improvement of Adolescent Parents in Family Legal Education*. Such program covered eight lectures on associated laws and regulations on the protection of minors, six lectures on methods of family legal education, i.e. *Tips on Children Education* (explanation with words, case discussion method, model demonstration method, evaluating analysis method, situation experiencing method and self-education method) and relevant discussions and interaction games.

### 3.2 Assessment on Execution Process of Education Intervention Program on Legal Cognition and Family Legal Education Methods for Adolescent Parents

We assessed the execution process of education intervention program mainly based on parents’ participation and satisfaction to know about the effectiveness of program execution. Parents filled in feedback sheet for unit activity after the completion of each activity, and filled in the questionnaire for overall assessment on activity after the completion of all activities. Data for analysis include activity records, parents’ feedback and knowledge learned and researcher’s field observation.

#### 3.2.1 Parents’ Participation

Ten parents participated throughout this activity, and parents’ participation was very high. “I have never participated in this kind of activity before, so I am very excited and bursting to participate.”

#### 3.2.2 Parents’ Satisfaction

1) Parents’ Feelings

We had provided parents with 15 words for their selection (they could select one or more), including 9 positive words and 6 negative words. No one selected the negative words.

From researcher’s observation, parents laughed happily and spoke and performed actively throughout this activity.

2) Group Atmosphere

In parents’ opinion, the group atmosphere was pleasant; the five-grade assessment index for 95% of parents was 5, and the average index was 4.95. “The atmosphere is pleasant, and if we fail to catch up with something, we can just raise questions.”

3) Time Control

All parents thought that the time was well controlled throughout the whole activities, and almost each activity was completed within the scheduled time. The five-grade assessment index for 85% of parents was 5, and the average index was 4.85.

4) Group Mentor

Parents highly appreciated the works of group mentor because the mentor could guide the activities effectively and obtain good teamwork of parents. The five-grade assessment index for 83.3% of parents was 5, and the average index was 4.83. “We share our own views, and the mentor finally comments on the views regardless of whether they are right or wrong. It gives face and also solves the problem. “

In conclusion, parents highly appreciated the execution process of the whole program and showed their interests in continual participation in education intervention activities.

### 3.3 Assessment on Results of Education Intervention Program on Legal Cognition and Family Legal Education Methods for Adolescent Parents

The purpose of assessment on results of group program is mainly to know about whether the manners of activities are appropriate, whether the contents of program are practical and feasible, whether positive changes have occurred in participants’ knowledge, attitudes and behaviors.

#### 3.3.1 Assessment on Manners of Activities

1) Time, Place and Name of Activities

Parents generally thought that the time, place and contents of activities selected met their actual needs and were with full consideration of parents.

2) Manners of Activities

Parents affirmed the new feelings brought to them by group activities, in which 83.3% like “role-play game” most and 50% like “story discussion” manner.

Role-play and group discussion increased the participation as parents have similar feelings in direct and stress free situations due to the nature that such activities imitate, and can comprehend the true meaning of various activities.

In parents’ opinion, feelings of rejection are easily evolved in adult learning if without favorable atmosphere. The relaxed and happy atmosphere in interaction and warm-up games shortened the gap among all members and broke the strangeness. “Playing games makes me feel much younger.”

Experience sharing among parents has improved their confidence in teaching the children. Upon the participation of group activity, some parents found they were not alone as other parents also had problems in teaching the children. “I am not worried about some problems anymore because they are common problems of children at this age.”

#### 3.3.2 Assessment on Contents of Activities

Parents thought that the group program was of high practicability and operability, as the saying goes “combination of knowledge and method is very effective”. Among all parents, 83.3% could fully understand such activities, and the average five-grade assessment index was 4.83.

#### 3.3.3 Assessment on Effectiveness of Activities

In the assessment on effectiveness of intervention activities, we not only considered parents’ assessment, but also added interviews with the children to get feedback information from them to support the effectiveness of activities.

1) Enrichment of Knowledge

In parents’ opinions, both their legal knowledge and legal education skills were enriched and improved after the education intervention activities. The five-grade assessment index for 66.7% of parents was 5, and the average index was 4.67. “The methods for educating children before are very dull and of poor effectiveness, but now we learn the methods creatively and apply them practically.”

2) Change of Attitudes

Both the parents and their children have felt positive changes in attitudes. The five-grade assessment index for 66.7% of parents was 5, and the average index was 4.67. “My father was always autocratic, but after that activity he told me he was surprised that there were so many rights of children to be protected and decided to communicate with me more frequently.”

3) Progress in Behaviors

Although it’s a short-term training and progress in behaviors is not that obvious compared with that in knowledge and attitudes, certain effectiveness has already been achieved. In terms of the timely transmission of activity contents to children, the five-grade assessment index was 5 for 50% of parents, and the average index was 4.50. In terms of the improvement of education methods and the application of new methods for practice, the five-grade assessment index was 5 for 66.7% of parents, and the average index was 4.67.

## 4. Analysis and Discussion

### 4.1 Assessment on Needs for Education Intervention Program on Legal Cognition and Family Legal Education Methods for Adolescent Parents

#### 4.1.1 Expectations on Activities

Parents were full of expectations on the training activities, in which they hoped to establish a new family education concept through learning legal education. According to the need & motivation theory of psychology, “need” is the basis of “motivation” and the source for individual’s dependence and positivity on objective conditions. Nothing else than parents’ expectations on activities endow them with strong motivation for participation in such activities.

#### 4.1.2 Manners and Contents of Activities

Time and place were set based on the characteristics of adult learning, and sensitive words in names were removed to release parents from stress. The activities were in group counseling manner. Such group counseling was flexible and diverse and could create a relaxed and harmonious atmosphere, in which parents could communicate and share with each other. Basic contents of activities were determined in accordance with anticipatory organizational goal and all opinions summarized.

### 4.2 Assessment on Execution Process of Education Intervention Program on Legal Cognition and Family Legal Education Methods for Adolescent Parents

The high participation of parents indicates that they could accept the group counseling manner, and had put their interests and enthusiasm into it. Adults “vote with their feet”, that is to say, that they feel terrible in the group or have no interests in the activities must be reflected in the attendance.

Parents felt well in and satisfied with the activities because the group counseling mentor could, with sincere and consensual attitudes, create a relaxed and acceptable psychological environment in which parents could open their hearts to comprehensively share what they had learned with other members. Special problems of individual parents were discussed after class, which reflected the respect to all members and allowed the whole group activities to be completed in a safe, reliable and harmonious atmosphere.

### 4.3 Assessment on Results of Education Intervention Program on Legal Cognition and Family Legal Education Methods for Adolescent Parents

#### 4.3.1 Assessment on Manners of Activities

In order to increase the number of participants, place and time for the implementation of parent education program must be convenient for parents. Jaffe’s research found that launching parent education activities at the time other than working hours will increase the participation of parents and can achieve significant effectiveness (Palm & Palkovitz, 1988; Fagan & Palkovitz, 2011). The time and place arrangement and names of this activity had met parents’ needs.

Parent education program is for adult education. Effective adult learning in groups could meet the needs of diversified learning, and at the same time build friendly interpersonal relationship; the activities are active and flexible, in which a support group of the same generation is formed, and it will exert a relatively long-term influence and changes (Levine, Pleck, & Lamb, 1983).

According to the social role theory (Zhang, 2008), role-play is the basis and means for role learning and role obtainment as well as the conditions for successful role achievement. In the situation created in the education intervention activities, parents, through actual role-play, recognized the role expectations on “qualified legal education parents”, learned the legal knowledge and legal education skills that needed in fact, and applied what they had learned into the child education practice, which reflected the effectiveness of “learning from practice”.

Activity approach takes knowledge concerning skills, emotions and practices as its main contents to promote overall development of the learner through the learner’s autonomous, open and creative practices under the guidance of the teacher. Learning legal norms and legal education methods must be combined with the main activities of learners (i.e. parents). Parents can master and apply them only after repeated practices. The open, autonomous and interactive games provided parents with a humorous and relaxed atmosphere, narrowed the gap between parents and removed the strangeness. In this way, parents’ emotions and reason could go together, and they could experience the knowledge in and contents of activities, which reflected the effectiveness of “learning from activities”.

According to the principles of cooperative learning (Pang & Wang, 2003), cooperative learning is a kind of activity under mutual help of peers, in which group activity is taken as its main parts. It emphasizes interaction among all members and common progress. The support and sharing in peer group among parents enabled them to feel that they had obtained various help in experience and support in emotions, which reflected the effectiveness of “learning from cooperation”.

#### 4.3.2 Assessment on Contents of Activities

The planning of parent education program takes the needs of target objects as the first factor for consideration because only the needs of target objects are met can those people with intention of participation be attracted (Peters & Day, 2000). The key is that the parents have the opportunity to make decisions; contents of such program are jointly decided by planners and learners. Before the planning of program, parents’ needs were investigated and needs assessment was conducted; during the execution of program, parents were made to express their needs for learning and needs recognized for improvement in legal cognition level and legal education skill, and accordingly, the program planner adjusted and modified the program in accordance with parents’ needs, and professionally guided and promoted the activities so that the parents could feel that attention were paid to them. The pertinence of learning process and the usefulness of learning contents helped them improve the level of child education.

#### 4.3.3 Assessment on Effectiveness of Activities

This education intervention activity witnessed the enrichment in parent’s legal knowledge, improvement in their legal education skills, and fully played functions of group counseling. The group counseling process is a process of self-development with the help of interaction among members. The members exchange information in the group, imitate each other and learn actively to improve their immature and deviant attitudes and behaviors and to promote self-acceptance and development (Liu, 2003)_._

## 5. Conclusion

Results have shown that, through the assessment of action research, the group training manner is a useful group consulting manner to make family legal education for adolescent parents. The program is feasible; the method is effective; the intervention effect is obvious.
